# Full-Length Transcriptome Analysis of the *ABCB*, *PIN/PIN-LIKES*, and *AUX/LAX* Families Involved in Somatic Embryogenesis of *Lilium pumilum* DC. Fisch.

**DOI:** 10.3390/ijms21020453

**Published:** 2020-01-10

**Authors:** Shengli Song, Zhiping Wang, Yamin Ren, Hongmei Sun

**Affiliations:** 1 Key Laboratory of Protected Horticulture of Education Ministry and Liaoning Province, College of Horticulture, Shenyang Agricultural University, Shenyang 110866, China; ssl_syau@163.com (S.S.); wangzp6119@163.com (Z.W.); RYM1258252544@163.com (Y.R.); 2 National and Local Joint Engineering Research Center of Northern Horticultural Facilities Design and Application Technology, Horticulture Department, Shenyang Agricultural University, Shenyang 110866, China

**Keywords:** full-length transcriptome, auxin transport, totipotency, somatic embryogenesis, *Lilium*

## Abstract

Plant cell totipotency is one of the 25 major topics in current scientific research, and somatic embryos are good experimental material for studying cell totipotency. Polar auxin transport plays an important regulatory role in somatic embryogenesis (SE). However, little is known about the auxin transport genes and their regulatory mechanisms in *Lilium* SE. In this study, we applied single-molecule real-time (SMRT) sequencing to *Lilium pumilum* DC. Fisch. for the first time and obtained a total of 119,649 transcripts, of which 14 encoded auxin transport genes. Correlation analyses between somatic embryo induction and gene expression under different treatments revealed that auxin transport genes, especially ATP-binding cassette (ABC) transporter B family member 21 (*ABCB21)* and PIN-FORMED (PIN) LIKES 7 (*PILS7)*, may be key players in SE, and the necessary duration of picloram (PIC) treatment to induce SE is as short as 3 days. Our research provides valuable genetic information on *Lilium pumilum*, elucidating the candidate auxin transport genes involved in SE and their influencing factors. This study lays a foundation for elucidating the regulatory mechanism of auxin transport in SE.

## 1. Introduction

Like animals, plants have complex mechanisms that regulate cell division, development and growth [1]. However, because plants cannot move in response to dynamic environmental conditions, most plant cells have strong developmental plasticity and can continuously regenerate and maintain totipotency [2]. Somatic embryogenesis (SE) is the developmental reprogramming of somatic cells toward embryogenesis. It is the embodiment of cell totipotency in higher plants [3]. Plant cells respond to different exogenous and endogenous signals that are involved in cell division, elongation, polarity, differentiation and totipotency induction. The transition from somatic to embryonic cells is the most pivotal process [4]. An efficient and synchronized somatic embryo regeneration system provides an important molecular basis for studying the transition from somatic to embryonic cells [5]. During this transition, dedifferentiation is needed to stimulate cell division and physiological, metabolic and gene expression [5,6].

Plant developmental plasticity relies on the ability of meristem to adapt to surrounding nutrients and environmental signals [7]. Among the various exogenous factors, plant growth regulators are the most widely used during the process of SE. Generally, most plant SE processes depend on stimulation by auxin at the preculture or induction stage [8,9,10,11]. Moreover, the ratio between auxin and cytokinin determines the fate of regenerative organs [12]. The distribution of auxin is determined mainly by auxin metabolism (synthesis, coupling and degradation) [13,14,15] and transport [16,17,18,19,20]. Free diffusion and active transport are the two main forms of auxin transport between cells. Under low pH conditions, auxin can enter cells by free diffusion after being protonated [21], while auxin transport is mainly based on active transport. Active transport requires not only the energy released by adenosine triphosphate (ATP) hydrolysis but also special transport carriers. For example, auxin can be transported to the cytosol through the action of AUXIN RESISTANT/LIKE AUXIN RESISTANT (AUX/LAX) family influx transporters [22]. In cells, auxin is negatively charged and therefore requires the aid of specific PIN-FORMED (PIN) and ATP-BINDING CASSETTE B-TYPE (ABCB) efflux transporters to enter the apoplast through the cell membrane [20]. PIN-LIKES (PILS) proteins may be involved in intracellular auxin transport between the cytosol and endoplasmic reticulum [23]. The endogenous auxin level also plays a very important role in SE [24]. If 2,4,6-trichlorophenoxyacetic acid, p-chlorophenoxyisobutyric acid, N-1-naphthyl phthalamic acid (NPA) or 2,3,5-triiodobenzoic acid (TIBA) [25,26] is added, abnormal somatic embryos will be produced, which also indicates that polar auxin transport plays a key role in the normal development process of somatic embryos [27].

*Lilium* spp. are horticultural plants with high economic and ornamental value in a great market demand [28]. However, owing to the absence of genomic information on *Lilium*, early studies on SE focused mainly on the application of plant growth regulators and the selection of explants in species such as *Lilium ledebourii* [29], *Lilium pumilum* [30], *Lilium longiflorum* [31] and *Lilium martagon* [32]. The process of *Lilium pumilum* SE has been reported [30]. Among the plant growth regulators, Pic is the most effective hormone in SE. SE mainly occurs via an endogenous pathway, with somatic embryos forming from the inner cells of embryogenic callus (EC). SE also occurs via an exogenous pathway, with somatic embryos developing from superficial cells or subsuperficial cells of EC. When the EC is transferred to a hormone-free medium, it can develop into a globular embryo, heart-shaped embryo, torpedo embryo and finally a complete plant [30]. In recent years, the function of microRNA (miRNA) in SE has been investigated, and it has been predicted and preliminarily verified that a large number of miRNAs play an important role in SE [28,33,34]. However, how fully differentiated explants regain totipotency and induce somatic embryos are still unclear.

Currently, auxin transport has been well studied in many plants, such as *Arabidopsis thaliana* [35], *Glycine max* [36], *Populus trichocarpa* [37], *Oryza sativa* [38], *Sorghum bicolor* [39], *Citrullus lanatus* [40] and *Zea mays* [41]. However, owing to the lack of genomic information on *Lilium*, auxin transport families have not been identified or studied in this genus, and studies considering auxin transport during SE have not been reported. With the emergence of full-length transcriptome sequencing technology, gene function studies in species without reference genomes have undoubtedly become more convenient. In this article, *Lilium pumilum* somatic embryos that originated in China were used for sampling and sequencing. The full-length transcriptome throughout the whole process of plant regeneration via SE was detected by the PacBio sequencing platform and the sequencing results were verified by second-generation sequencing (SGS). Gene cloning was carried out to verify the accuracy of the reference transcriptome library. To study the molecular mechanism of SE, the members of the ABC family, PIN/PILS family, and AUX/LAX family were predicted for the first time in *Lilium pumilum.* The members of the ABCB subfamily, PIN/PILS family and AUX/LAX family, which participate in polar auxin transport were identified and screened, and 14 genes involved in polar auxin transport were cloned for the first time in *Lilium pumilum*. The comparison of the expression patterns under different conditions indicated that *ABCB21* and *PILS7* have important regulatory roles in SE. This article provides a relatively complete and accurate reference transcriptome for SE or even other biological processes of *Lilium pumilum* and preliminarily confirms that water stress may be one of the reasons why somatic cells regain totipotency, while polar auxin transport promotes the development of totipotent cells into somatic embryos.

## 2. Results

### 2.1. Effects of External Environmental Factors on SE

As shown in Table 1, whether in light or darkness, somatic embryos were observed when scales were cultured in Murashige-Skoog (MS) media supplemented with 1.0 mg/L picloram (PIC) for different treatment durations and then subcultured in hormone-free media for 50 days. However, the induction rate of somatic embryos reached 80.0% in light but only 33.3% in darkness when PIC was applied for 3 days. The induction rate of somatic embryos reached 100% when PIC was applied for 12 days in light, while in darkness, treatment for 18 days was needed for induction. In light, with the increase in PIC treatment duration, the induction rate of adventitious buds gradually decreased, and the induction rate of adventitious roots showed no obvious trend. In darkness, with the increase in PIC treatment duration, the induction rate of adventitious roots gradually increased, and the induction rate of adventitious buds showed no obvious trend. In addition to a small amount of adventitious buds production in light conditions, PIC treatment in light or darkness for 50 days resulted in the production of only ECs.

**Figure 1:**
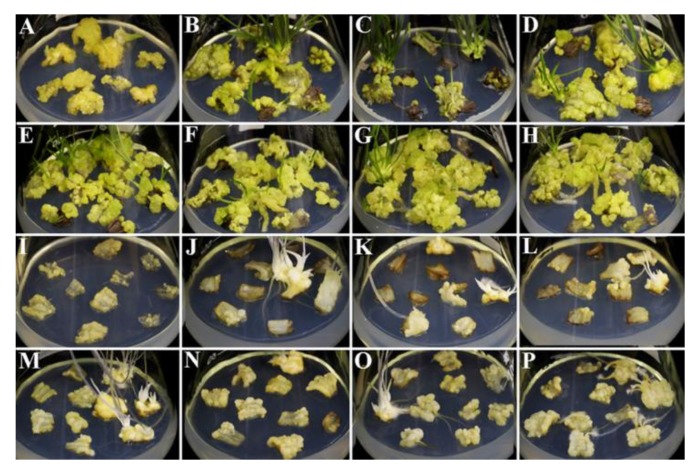
Somatic embryo and adventitious bud induction (50 days). (**A**,**I**) were cultured in Murashige-Skoog (MS) media supplemented with 1.0 mg/L PIC throughout the experiment. (**B**–**H**,**J**–**P**) were cultured in MS media supplemented with 1.0 mg/L PIC for 3, 6, 9, 12, 15, 18 and 21 days and then subcultured into MS media without hormones for 50 days. (**A**–**H**) show light culture; (**I**–**P**) show dark culture.

As shown in Figure 1, compared with culture in darkness, light culture resulted in more and larger ECs, and light culture also increased the induction rate of adventitious buds. In light, EC showed a light-yellow in color when treated with 1.0 mg/L PIC for 50 days, while EC showed a light-green in color when treated with PIC for 3–21 days.

**Figure 2:**
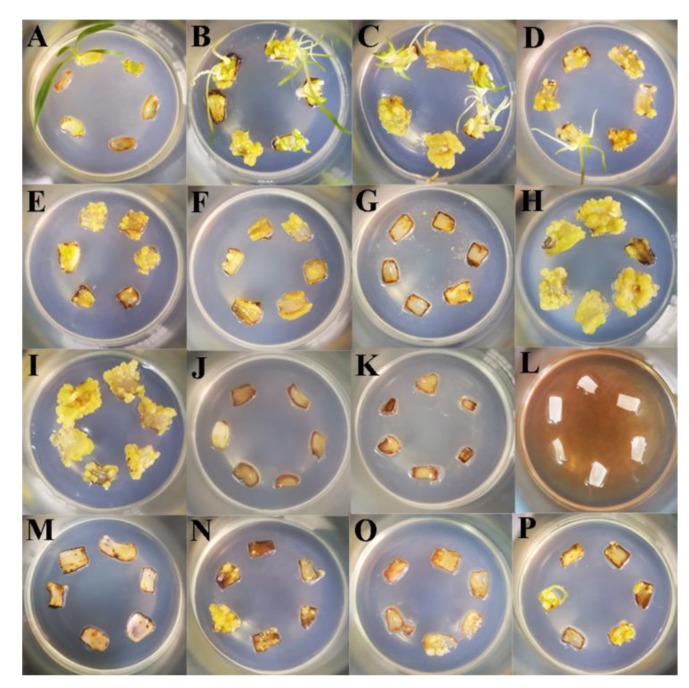
The effects of different treatments on somatic embryo induction (50 days). Explants were inoculated in the following media for 50 days: (**A**) MS media; (**B**–**G**) MS media supplemented with PIC at 0.5, 1.0, 2.0, 3.0, 4.0 and 5.0 mg/L, respectively; (**H**–**L**) MS media supplemented with PIC at 1.0 mg/L with the pH adjusted to 4.8, 5.8, 6.8, 7.8 and 9.8, respectively; (**M**) MS media without macroelements but supplemented with PIC at 1.0 mg/L; (**N**) MS media without microelements but supplemented with PIC at 1.0 mg/L; (**O**) MS media without iron salts but supplemented with PIC at 1.0 mg/L; (**P**) MS media without organic substances but supplemented with PIC at 1.0 mg/L; All the media were adjusted to pH = 5.8 with 1 M NaOH except (**H**) and (**J**–**L**).

As shown in Table 2 and Figure 2, the explants cultured in MS media (M) produced only a few adventitious buds or calli. However, the induction rate of calli or adventitious buds increased when the MS media were supplemented with different concentrations of PIC (P1–P6). The callus induction rate first increased but then decreased when the PIC ranged from 0.5–5.0 mg/L and reached 100% when the PIC was 1.0 mg/L (P2). The number of adventitious buds decreased as the concentration of PIC increased, and there were no adventitious buds when the PIC was greater than 3.0 mg/L (P4–P6). The pH of the media had a significant effect on SE when the PIC was 1.0 mg/L. When the pH of the media was 4.8 (pH1) or 5.8 (pH2), the induction rate of ECs reached 94.4% or 100%, respectively. As the alkalinity increased, little amounts of calli were produced (pH3–pH5). To verify the effects of nutrient elements on SE, somatic embryo induction was carried out without macroelements, microelements, iron salts or organic substances at pH = 5.8 and PIC = 1.0 mg/L. The results showed that MS media without macroelements sharply reduced the induction rate of somatic embryos (A1) and that media without microelements, iron salts or organic substances had a weak effect (A2–A4).

### 2.2. Comparison of Transcripts between Iso-seq and RNA-seq

**Figure 3:**
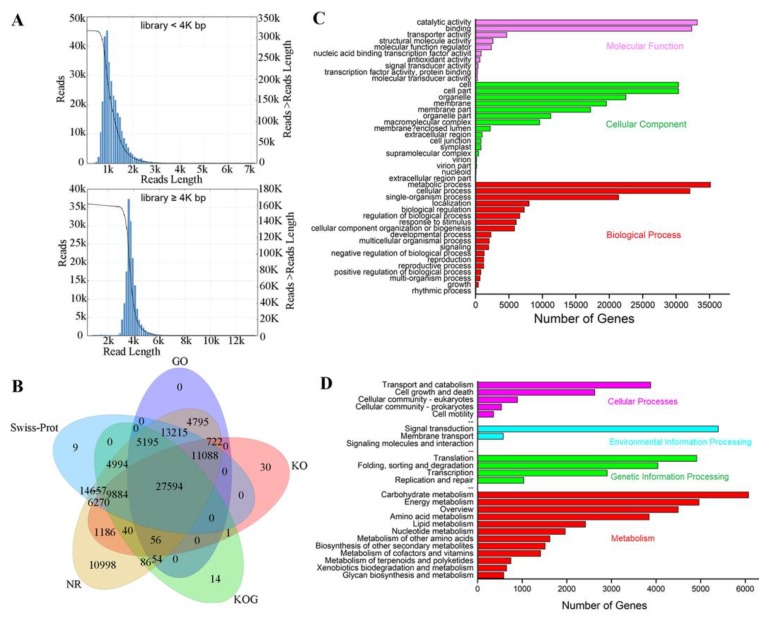
Analysis and functional annotation of full-length transcriptome data. (**A**) Full-length nonchimeric (FLNC) sequence length list. (**B**) The transcripts were annotated via five databases, and the results are displayed in a Venn diagram. (**C**) Gene Ontology (GO) enrichment analysis. (**D**) Kyoto Encyclopedia of Genes and Genomes (KEGG) enrichment analysis.

The sequences produced by the third-generation PacBio sequencing platform were ultimately obtained as Iso-seq (Isoform-sequencing) sequences after quality control filtration, error correction and redundancy removal. The previous RNA-seq transcripts were used as controls. The sequence fragments of the Iso-seq were relatively long, but the number of transcripts was somewhat reduced (Table S1). The maximum sequence length from Iso-seq was approximately 6 kb (Figure 3A).

### 2.3. Functional Annotation of Iso-seq Transcripts

Five databases were used for isoform functional annotation: The National Center for Biotechnology Information (NCBI) nonredundant protein sequences (NR), Gene Ontology (GO), euKaryotic Ortholog Groups (KOG), Kyoto Encyclopedia of Genes and Genomes (KEGG), and Swiss-Prot protein sequence databases. Among the 119,649 nonredundant isoforms obtained, 110,888 (92.68%) were successfully annotated in at least one of the five databases after Basic Local Alignment Search Tool (BLAST) searches were performed, and 27,594 were functionally annotated in all five databases (Figure 3B). On the basis of the KEGG annotation map (Figure 3D), a large number of isoforms were involved in the process of carbohydrate metabolism (6072) and signal transduction (5396), which corresponded to the metabolic process (35,123) and catalytic activity (33,163) in the GO terms annotation (Figure 3C), implying that carbohydrate metabolism and signal transduction play an important role in *Lilium pumilum* SE.

### 2.4. Identification and Analysis of Family Members Involved in Polar Auxin Transport

To identify the gene families involved in polar auxin transport, HMMER3.1 was used to search the conserved domains of members of the ABC family, PIN family and AUX/LAX family in the Pfam database. The obtained protein sequences were further analyzed by NCBI BLASTp to exclude the protein sequences lacking the domain. Sixty-three ABC family members were ultimately predicted in *Lilium pumilum*; among the subfamilies, the ABCG subfamily members were predicted the most predominant (20), followed by ABCB subfamily members (15). The proteins had lengths ranging from 253 aa (LpABCA12) to 1913 aa (LpABCA1), molecular weights (MWs) ranging from 28.16 kd to 213.75 kd, and isoelectric points (pIs) ranging from 5.37 to 9.99. Eleven members of the PIN/PILS family (seven PINs and four PILSs) with amino acid lengths of 221 aa (LpPIN5) to 651 aa (LpPIN3A), MWs of 24.23 kd to 69.88 kd, and pIs of 6.13 to 9.76, were predicted. Moreover, it was predicted that there were 23 amino acid/polyamine transporter 2 family members, including three AUX/LAXs, and their amino acid lengths ranged from 212 aa (LpAvt6B) to 530 aa (LpAvt1A), their MWs ranged from 22.66 kd to 57.67 kd and their pIs ranged from 4.88 to 9.59 (Table S2).

### 2.5. Genetic Analysis of Members of the Polar Auxin Transport Family

**Figure 4:**
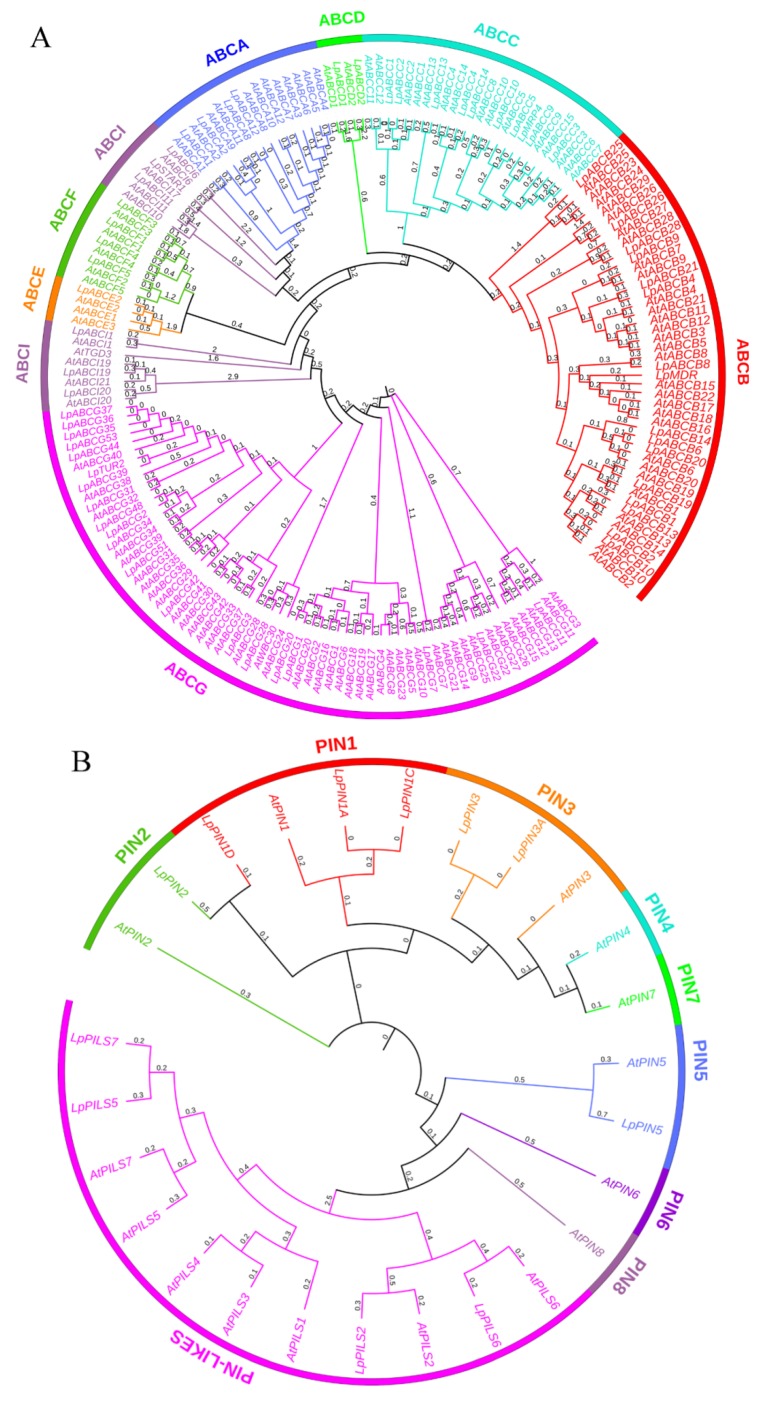

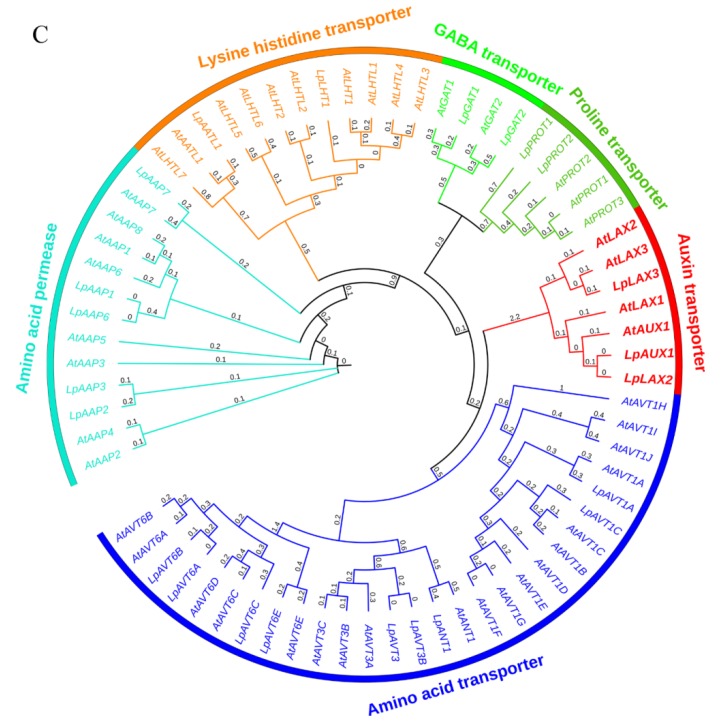
Phylogenetic relationships of members of the ATP-binding cassette (ABC), PIN/PILS and AUX/LAX families in *Lilium pumilum* (Lp) and *Arabidopsis thaliana* (At). (**A**) ABC family; (**B**) PIN/PILS family; (**C**) AUX/LAX family.

The protein sequences of 120 ABC transporter superfamily members, 15 auxin efflux carrier family members and 46 amino acid/polyamine transporter 2 family members of *Arabidopsis thaliana* were downloaded from the UniProtKB/Swiss-Prot database (Reviewed). Multiple Alignment using Fast Fourier Transform (MAFFT) online software was then used to compare the amino acid sequences of the members of the three families identified in *Lilium pumilum* with those of *Arabidopsis thaliana*, after which FastTree software was used to construct an evolutionary tree. As shown in Figure 4, the ABC family genes were divided into eight subfamilies; ABCG subfamily members were predominant (63), followed by the ABCB subfamily members (45). There were at least four ABCD and ABCE subfamily members, and ABCI subfamily members were separated by the ABCE and ABCF subfamily. Because the amino acid/polyamine transporter 2 family members were found to have an Aa_trans (PF01490) domain, 46 *Arabidopsis thaliana* members and 23 *Lilium pumilum* members were selected to construct an evolutionary tree, which was divided into six categories, which included seven auxin transporter members. By domain search, the members of the PIN/PILS family were predicted to have the least number of protein sequences—only eleven (seven PINs and four PILSs).

### 2.6. Conserved Domain Analysis of Members of the Polar Auxin Transport Family

**Figure 5:**
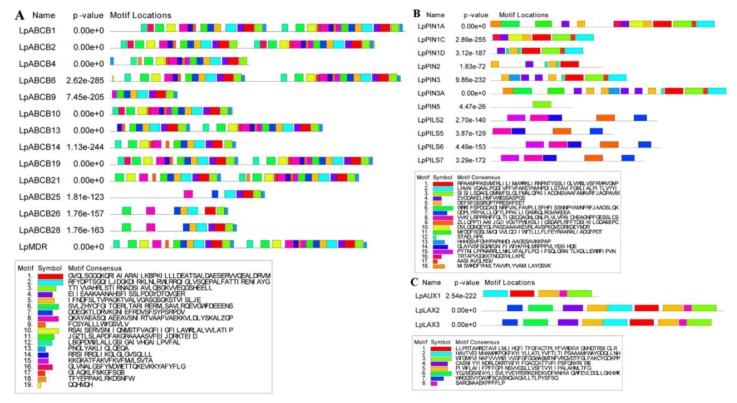
Motif analysis of fourteen LpABCBs (**A**), eleven LpPINs/PILSs (**B**) and three LpAUX/LAXs (**C**).

The conserved domains of 14 ABCBs, 11 PINs/PILSs and three AUX/LAXs were analyzed via Multiple Em for Motif Elicitation (MEME) online domain analysis software, and a motif with an E-value larger than 0.05 was identified (Figure 5). According to the ABCB subfamily in *Arabidopsis thaliana*, AtABCB1-AtABCB23 are full-sized transporters (two nucleotide-binding domains (NBD) and two transmembrane domains (TMD)), and AtABCB24-AtABCB29 are half-sized transporters (one NBD). LpABCB1, LpABCB2, LpABCB6, LpABCB19, LpABCB21 and LpMDR have similar domains. Moreover, the first half and the second half of each protein are very similar, and all of these proteins are full-sized transporters. However, LpABCB4, LpABCB9, LpABCB10, LpABCB13 and LpABCB14 do not have a complete full-sized transporter structure. LpABCB25, LpABCB26 and LpABCB28 are half-sized transporters, but only LpABCB25 has a complete structure. Similarly, compared with their homologs in *Arabidopsis thaliana*, LpLAX2, LpLAX3, LpPIN1A, LpPIN3A, LpPILS2, LpPILS6 and LpPILS7 have similar and complete conserved domains, and LpLAX2 and LpLAX3 are highly similar.

### 2.7. Cloning and Verification of Genes Involved in Polar Auxin Transport

**Figure 6:**
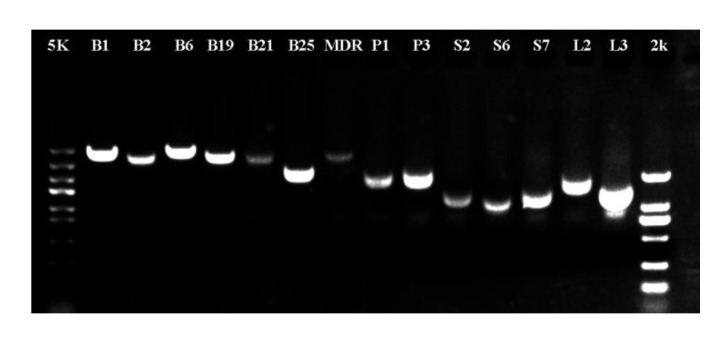
Full-length cDNA cloning of genes involved in polar auxin transport. 5 k: 5 kb marker; 2 k: 2 kb marker. B1, ABCB1; B2, ABCB2; B6, ABCB6; B19, ABCB19; B21, ABCB21; B25, ABCB25; MDR, MDR; P1, PIN1A; P3, PIN3A; S2, PILS2; S6, PILS6; S7, PILS7; L2, LAX2; L3, LAX3.

To verify the accuracy of the third-generation sequencing data, fourteen genes that are involved in polar auxin transport and that have full-length open reading frames (ORFs) were chosen for cloning. The primers used for gene cloning are listed in Table S3. Fourteen genes were ultimately successfully cloned, and their fragment lengths were in line with our expectations (Figure 6).

### 2.8. Expression Pattern Analysis of Genes Involved in Polar Auxin Transport

Specific gene primers for quantitative real-time PCR (qRT-PCR) were designed according to the gene sequences. The specificity and amplification conditions of the primers were screened. Specific primers and their corresponding midpoint temperatures (TMs) were ultimately obtained (Table S4).

**Figure 7:**
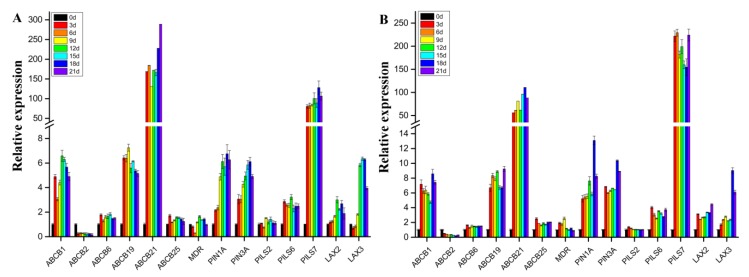
Expression pattern analyses of 14 genes involved in polar auxin transport in somatic embryo induction media under darkness (**A**) or light (**B**) for different durations.

As shown in Figure 7, whether in light or darkness, in addition to *ABCB2*, the expression levels of the other thirteen genes involved in polar auxin transport presented different degrees of upregulated patterns with different treatment durations. The expression of nine of the thirteen genes was significantly upregulated. Among these genes, the expression patterns of *ABCB21* and *PILS7* were the most significantly different. In light and darkness, the expression level of *ABCB21* was upregulated by approximately 79 times and 191 times, respectively, and that of *PILS7* was upregulated by approximately 96 times and 196 times, respectively. The expression level of *ABCB21* increased overall with increasing treatment duration. However, *PILS7* expression increased in darkness, but decreased in light. The expression of *ABCB1*, *ABCB19*, *PIN1*, *PIN3*, *PILS6*, *LAX2* and *LAX3* was upregulated by approximately 2–8 times. However, there were no significant changes in the expression of *ABCB6*, *ABCB25*, *MDR,* or *PILS2*. The expression of only one gene, *ABCB2*, was significantly downregulated. A comparison of Figure 7A,B shows that light culture conditions promoted the expression of eleven genes involved in polar auxin transport but did not promote the expression of *ABCB6*, *ABCB21* or *PILS2*.

**Figure 8:**
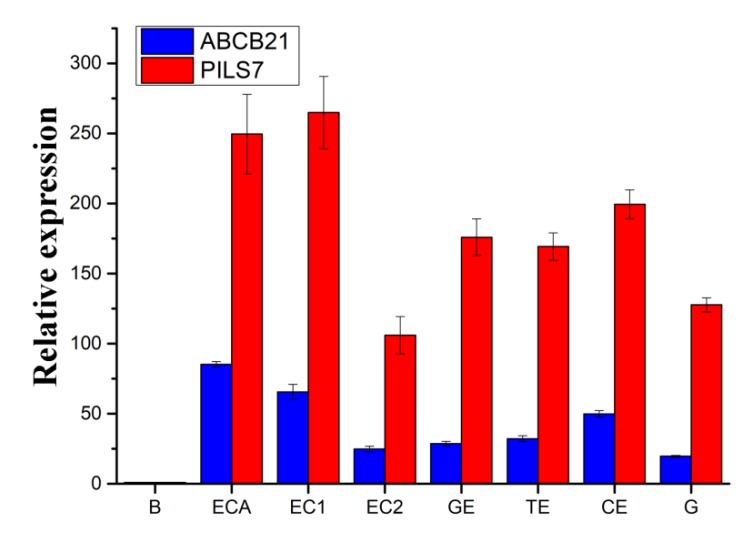
The expression levels of *ABCB21* and *PILS7* at different stages of somatic embryos. B, scales; ECA, embryonic cells appear; EC1, early proembryo; EC2, late proembryo; GE, globular embryo; TE, torpedo embryo; CE, cotyledon embryo; G, germination.

To analyze the mechanism of *Lilium pumilum* SE. *ABCB21* and *PILS7*, whose expression most obviously differed, were selected to analyze the expression patterns. When somatic embryos showed a distinct phenotype at the induction stage, the expression levels of *ABCB21* and *PILS7* were upregulated approximately 85 times and 250 times, respectively (Figure 8, ECA). The expression level of *ABCB21* was downregulated, while that of *PILS7* was upregulated at early proembryo stage (Figure 8, EC1). At the germination stage, the expression levels of *ABCB21* and *PILS7* were significantly downregulated (Figure 8, EC2) and then gradually increased until cotyledon embryo was produced (Figure 8, GE-CE). When somatic embryos germinated, the expression levels of *ABCB21* and *PILS7* reached a relatively low level (Figure 8, G).

**Figure 9:**
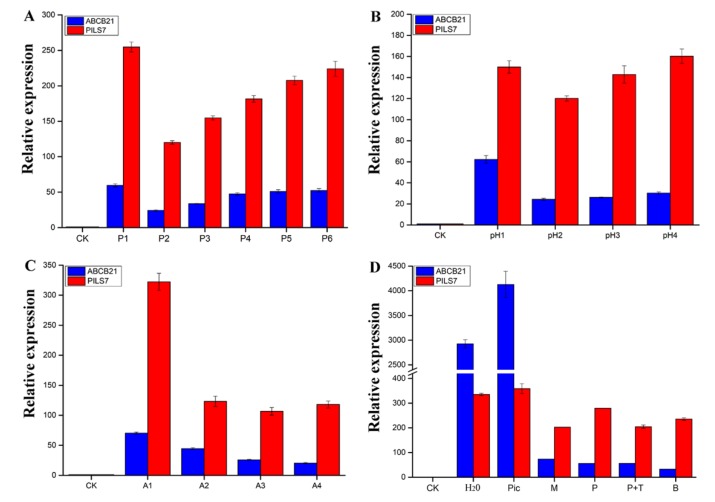
Expression pattern analysis of *ABCB21* and *PILS7* under different treatments for 3 days. (**A**) The expression of *ABCB21* and *PILS7* under different concentrations of PIC; (**B**) The expression of *ABCB21* and *PILS7* under different pH; (**C**) The expression of *ABCB21* and *PILS7* under different media compositions; (**D**) The expression of *ABCB21* and *PILS7* under different hormones. CK, untreated scales; H_2_O, water; Pic, water supplemented with PIC at 1.0 mg/L; B, MS media supplemented with 6-BA at 1.5 mg/L; P+T, MS media supplemented with PIC at 1.0 mg/L and 2,3,5-triiodobenzoic acid (TIBA) at 1.0 mg/L; M, MS media; P, MS media supplemented with PIC at 1.0 mg/L; P1–P6, MS media supplemented with PIC at 0.5, 1.0, 2.0, 3.0, 4.0 and 5.0 mg/L, respectively; pH1–pH4, MS media supplemented with PIC at 1.0 mg/L, with the pH adjusted to 4.8, 5.8, 6.8 and 7.8, respectively; A1, MS media without macroelements but supplemented with PIC at 1.0 mg/L; A2, MS media without microelements but supplemented with PIC at 1.0 mg/L; A3, MS media without iron salts but supplemented with PIC at 1.0 mg/L; A4, MS media without organic substances but supplemented with PIC at 1.0 mg/L; All the media were adjusted to pH = 5.8 by 1 M NaOH except pH1 and pH3-pH4.

As shown in Figure 9, the expression trends of *ABCB21* and *PILS7* were similar under different concentrations of PIC, different medium pH values, and different medium compositions. Compared with those of the control (Figure 9D, CK), the expression levels of *ABCB21* and *PILS7* after 3 days of treatment with water were highly upregulated approximately 2925 times and 335 times, respectively (Figure 9D, H_2_O), and PIC further promoted their expression up to approximately 4127 times and 358 times, respectively (Figure 9D, Pic). However, the culture of scales in MS media resulted in decreased expression of both genes, especially *ABCB21* (Figure 9D, M), and the culture of scales in MS media supplemented with different concentrations of PIC (Figure 9A, P1–P6) resulted in reduced expression of both genes, with the lowest expression at 1.0 mg/L PIC (Figure 9A, P2). The expression level subsequently gradually increased with increasing PIC concentration. The PIC and pH treatments revealed similar patterns. When pH = 5.8, the expression of *ABCB21* and *PILS7* was the lowest (Figure 9B, pH2), and pH increased within a certain range increased the expression of both genes. When pH = 4.8, the expression of *ABCB21* was the highest (Figure 9B, pH1); however, when pH = 7.8, the expression of *PILS7* was the highest (Figure 9B, pH4). Use of the different medium compositions revealed that the expression levels of *ABCB21* and *PILS7* were highest without the addition of macroelements (Figure 9C, A1), and the levels were slightly greater than those under culture with MS medium alone (Figure 9D, M). The expression of *ABCB21* was the lowest without the addition of organic substances (Figure 9C, A4), while that of *PILS7* was the lowest expression without the addition of iron salts (Figure 9C, A3). The use of the different hormone treatments indicated that *ABCB21* and *PILS7* presented different response trends. Compared with the results when MS media without the addition of hormones were used (Figure 9D, M), *ABCB21* expression was inhibited and *PILS7* expression was promoted after treatment with 1.0 mg/L PIC or 1.5 mg/L 6-Benzylaminopurine (6-BA) for 3 days (Figure 9D, P, B). Moreover, the expression level of *ABCB21* was nearly the same after the addition of 1.0 mg/L TIBA, while that of *PILS7* decreased (Figure 9D, P+T).

## 3. Discussion

Since the emergence of RNA-seq, model plants and non-model plants such as Arabidopsis [42], rice [43] and maize [44] have been sequenced. However, owing to incomplete and low-quality RNA-seq results, it is impossible to annotate all transcripts accurately [45]. Third-generation sequencing technology was performed at the single-molecule level for real-time detection of DNA sequences. Owing to its long reads, high sensitivity, lack of GC bias and ability to directly detect modified bases, single-molecule real-time (SMRT) sequencing has solved several major problems associated with RNA-seq. High-quality and full-length transcripts are directly obtained from the 5’ to 3’ direction, and the transcripts do not require assembly, both of which surpass the limitations of RNA-seq of partial cDNA sequences. Moreover, complex rapid amplification of cDNA ends (RACE) technology is not needed for full-length cDNA cloning, especially for long gene fragments. The advantages of third-generation sequencing technology are more obvious. Third-generation sequencing has great advantages in terms of the recognition of transcripts of isoforms, homologous genes, superfamily genes and in terms of allele expression [46,47]. However, recent studies have shown that third-generation sequencing can produce inaccurate genetic information, leading to an increased error rate [48]. Owing to the short sequence fragments obtained with RNA-seq, the single sequence fragments obtained from this method are relatively more accurate. Therefore, full-length transcripts obtained with third-generation sequencing can be proofread with RNA-seq to obtain complete genetic information at the transcriptional level [49]. In the present study, compared with the RNA-seq data, in which the average transcript length was 761 bp, the third-generation sequencing fragments, whose average length was 2017 bp, were more complete. Third-generation sequencing is also more accurate than RNA-seq with respect to transcript annotation. Our results showed that unannotated transcripts constituted only 7.32%. Thus, novel genes likely exist because *Lilium* is an ancient taxon, and the unannotated genes may have novel functions. The full-length cDNA sequences of fourteen genes were successfully cloned by querying the sequence information of the third-generation transcriptome, indicating that we provided an accurate reference genome for studies on SE of *Lilium pumilum* and even for other *Lilium* species or for other biological processes.

SE requires cells to respond to different exogenous and endogenous signals [4]. Carbohydrates can serve as energy and carbon sources, and they also act as important osmotic agents and signaling molecules in plants [50]. Some metabolites are associated with either normal or aberrant embryo development [51], and specific carbohydrates may play an important role in different steps of SE development [52]. Research in Arabidopsis has shown that carbohydrates play a role in the G2/M transition in cells via sugar signaling, which directly affects the proliferation of meristematic tissue [53]. A study in *Pinus pinaster* also reveals upregulation expression of genes involved in carbohydrate transport and metabolism in early stages of embryogenesis but downregulation expression toward the later stages of embryogenesis [54]. GO and KEGG pathway annotation analyses also revealed that many genes were involved in carbohydrate metabolism and signal transduction. Carbohydrates have multiple functions in plant development and it is difficult to distinguish between these functions, especially between their roles as an energy source and signaling molecule [52]. It has been shown that enhanced sucrose availability positively affects SE yields [52].

Although members of the amino acid/polyamine transporter 2 family have an amino acid transporter (Aa_trans) domain (PF01490), only four AUX/LAXs are involved in auxin transport [55,56,57]. The numbers of the three family members predicted in *Lilium pumilum* were significantly lower than those in *Arabidopsis thaliana*, which was mainly due to the insufficient the sequencing depth for the large genome of *Lilium* and possibly occurred because only the whole SE process was used for sequencing. The unpredicted members may not participate in SE, but the predicted members may have a certain function in SE, laying the foundation for elucidating the mechanism of *Lilium pumilum* SE. Although the number of auxin transporter genes predicted in *Lilium pumilum* was small, it was very similar to that in *Arabidopsis thaliana* in some respects. For example, the ABC transporter superfamily of *Lilium pumilum* could also be divided into eight subfamilies, and no ABCH subfamily members were predicted, as is the case for other plant species [58,59]. With the exception of LpMDR, which was more homologous to a gene in rice, the genes of all ABC members were most similar to genes in *Arabidopsis thaliana*.

To study the key external environmental factors that affect SE, scales were cultured in MS media containing PIC for different durations and then subcultured in MS media without the addition of any hormones in light and darkness. In light or darkness, scales cultured in MS media without any hormones gradually turned brown and died, but because of differences in the status and hormone contents of the scales, a few scales formed a very small number of adventitious buds, adventitious roots or calli (Figure 2A). Mikuła also reported that somatic embryos of ferns could be induced in the absence of plant growth regulators [60]. Compared with fern SE, *Lilium* SE is more complex and difficult, and it generally requires treatment with exogenous hormones. Somatic embryos are more likely to be induced when the explants contained higher endogenous hormone levels [61]. Auxin is usually synthesized in the shoot apex and the developing leaf primordia, and then transports to the targeted tissues by bulk flow via vascular tissues or polar transport [22]. This also suggests that explants are not provided exogenous hormones may undergo regeneration. However, there is an important relationship between the type and concentration of exogenous hormones and the content of endogenous hormones in explants capable of undergoing regeneration when treated only with exogenous hormones. The use of exogenous plant hormones in vitro can enhance plant regeneration ability [12]. In the present study, a short duration (3 days) of PIC treatment in light achieved an EC induction rate of 80.0%, while in darkness, the induction rate was only 33.3%. However, light promoted the production of increased numbers of adventitious buds, which indicates that light promotes EC polarity formation and induces adventitious buds generation during *Lilium pumilum* SE (Table 1). These findings also indicate that exogenous plant hormones are key factors in the differentiation direction of explants and that light can both promote and inhibit the differentiation direction of explants in different regeneration processes. The process of auxin transport is very fast [35]; thus, PIC treatment for less than 3 days may promotes SE. However, PIC treatment promoted only EC regeneration and inhibited adventitious buds and adventitious roots regeneration. Therefore, light may promote the regeneration process, and PIC may inhibit organ polarity formation. Combined with the expression results, the results showed that light inhibited *ABCB21* expression levels and that increased PIC treatment duration increased *ABCB21* expression levels, while light and increased PIC treatment duration had a synergistic effect on *PILS7,* that is, *PILS7* expression levels increased with the prolonging of PIC treatment duration in darkness; however, in light, the opposite was true. Moreover, the expression levels of *ABCB21* and *PILS7* in SE also show that *ABCB21* and *PILS7* may play an important role in regulating SE (Figure 8 and Figure 9), but further studies are needed to identify the exact underlying mechanism.

Plant developmental plasticity relies on the ability of the meristem to adapt to surrounding nutrients and environmental signals [7]. Explants depend not only on their own nutritional status but also on appropriate environmental conditions to undergo regeneration during in vitro culture. Therefore, differences in the nutrient status, endogenous hormone contents and optimal growth conditions of different explants may be the main reasons for various plant regeneration requirements in vitro, including different concentrations and types of hormones and different growth conditions. The results of our experiments showed that different concentrations of PIC, the pH of the medium and the components of nutrient elements played an important role in SE.

A previous report showed that low pH could promote auxin entry into cells by free diffusion [21], and the acid-growth hypothesis also indicated that acidic conditions were beneficial to plant development [62,63]. In a recent study in sugar cane, adenosine triphosphatase (ATPase) related proteins were found to be more abundant during somatic embryo maturation [64]. Additionally, somatic embryo maturation increased with the differential accumulation of proteins associated with H^+^ flux [65]. Changes in H^+^ flux have been reported in embryogenic and nonembryogenic calli, which presented a high H+ influx during somatic embryo formation [66]. These results indicate that H^+^ flux is an important factor during SE. Moreover, H^+^ influx is also thought to be associated with the maintenance of embryogenic competence in *Daucus carota* calli [67]. H^+^ flux can also affect the intracellular and extracellular pH. The intracellular pH is closely related to the transition from somatic to embryogenic cells [68]. Callus cells having a relatively acidic vacuole are an important characteristic of embryogenic competence [66]. Our results were consistent with those of previous studies, and somatic embryos occurred mostly under acidic conditions only and not under neutral or alkaline conditions (Figure 2H–L). Acidic conditions may be a requirement of auxin function, which also depends on the optimum pH of different plants at the same time. Interestingly, the expression levels of *ABCB21* and *PILS7* were lowest at pH = 5.8, which was the best pH for SE. According to the acid-growth hypothesis, we speculate that the pH may alter the direction of auxin transport.

At present, there are many synthetic auxin analogues. Among them, 2,4-dichlorophenoxyacetic acid (2,4-D) is the most commonly used one in SE, but PIC is more effective for *Lilium* SE. In this experiment, we found that the addition of PIC promoted the expression of *ABCB21* and *PILS7*. The expression of *ABCB21* and *PILS7* tended to decrease but then gradually increased when PIC was supplied within the range of 0.5–5.0 mg/L (Figure 9A). When the PIC was supplied at 1.0 mg/L, the induction rate of the somatic embryos was greater than that at other concentrations (Figure 2B–G). Since ABCB21 has been proven to be a two-way transport carrier, when the concentration of exogenous auxin is low, auxin is transported outward and vice versa [69,70]. Therefore, we speculate that, like *ABCB21*, *PILS7* employs two ways to transport auxin; that is, *PILS7* couples auxin when it is at low concentrations and releases auxin when it is at high concentrations. The endogenous auxin content of different species and different explants will also affect the direction of auxin transport. Although *ABCB21* [69,70] and *PILS7* [23] are expressed in different tissues, their expression patterns are very similar, and it is likely that *ABCB21* and *PILS7* jointly participate in auxin signaling to regulate plant growth and development.

Pedroso and Pais reported that a high level of inorganic elements (Ca, K, Na, Mg, S, P and Fe) might be related to embryonic development or morphogenesis in *Camellia japonica* [71,72,73]. The calcium signaling pathway combined with stress and endogenous hormone-related proteins plays an important role in somatic embryo induction and germination, and the addition of CaCl_2_ can promote callus induction and somatic embryo germination in banana [74]. Our results showed that the absence of one of the four groups of elements had a negative effect on SE and that macroelements were the most important. The expression levels of *ABCB21* and *PILS7* were significantly inhibited when macroelements were added, and the induction rate of somatic embryos was slightly reduced; however, the expression levels of *ABCB21* and *PILS7* were significantly increased in the absence of macroelements, but somatic embryo induction rate was significantly reduced. However, the absence of one of the four groups of elements inhibited SE (Figure 2). Moreover, nutrient elements had a significant inhibitory effect on *ABCB21* expression but not on *PILS7* expression, which means that nutrient elements may inhibit the influx of exogenous auxin or the efflux of endogenous auxin. This indicates that explants with high endogenous auxin contents may spontaneously form somatic embryos under adequate nutrient conditions. Therefore, apart from inorganic elements [71,72,73], all of the nutrients with suitable concentrations promoted SE. Additionally, the observed high H^+^ influx might be associated with high carrier transporter activity, especially calcium and carbohydrate transporters, which are relatively abundant in ECs [66]. Therefore, we speculate that inorganic elements, especially calcium, may participate in the auxin transport process.

The expression levels of *ABCB21* and *PILS7* were reduced after the addition of PIC, PIC+TIBA or 6-BA, and their expression patterns differed. The expression of *ABCB21* was nearly unchanged, while the expression of *PILS7* was downregulated in response to TIBA addition, and SE was completely suppressed. These findings indicate that *PILS7* may have a decisive role in SE and that *ABCB21* can promote SE. The PILS protein promoted the accumulation of indole-3-acetic acid (IAA), but did not affect the accumulation of 6-BA [23]. Moreover, cytokinin inhibited the expression of *ABCB21* [35,75,76]. We also found that the expression of *ABCB21* decreased after the addition of 6-BA, while that of *PILS7* increased. This may have occurred because *ABCB21* is not a receptor for 6-BA, and *PILS7* promotes auxin metabolism in the presence of excess cytokinin to balance intracellular auxin and cytokinin levels. Since the ratio between auxin and cytokinin determines the development direction of organs, we speculate that the balance between auxin and cytokinin indirectly influences the expression of *PILS7* and *ABCB21*.

Plant regeneration via SE is highly genotype dependent [74,77]. Moreover, the growth state of each explant is divergent, thus, the phenotype can be different under the same external environment. Rose believed that the formation of totipotent cells can be induced under the joint action of explants, stress and hormones [78]. In this experiment, it was also found that the induction efficiency of different explants under the same culture conditions differed (Figure 1 and Figure 2), which indicates that the type and growth status of explants determine the degree of difficulty in SE and that SE is regulated by the joint action of stress and exogenous hormones. Although the mechanism of auxin and stress-induced SE is still unclear, the biosynthesis of endogenous auxin induced by auxin and stress is considered to be the key process in the early transition from somatic cells to totipotent cells [79].

The relationship between stress and SE is currently attracting increasing attention. It has been found that many genes related to SE are involved in stress signaling pathways [80]. Totipotent cells can be formed by the dedifferentiation of somatic cells, which are induced by the addition of certain plant hormones, culture under different stress conditions or their simultaneous application [81]. For example, high temperatures increase auxin signaling [82] and significantly reduce the content of available water [83]. Since temperature stress can promote the regeneration process of cells, raising or lowering the temperature may promote somatic embryo initiation and proliferation [84]. Some studies have suggested that the interactions between abscisic acid (ABA) and auxin play an important role in plant growth and development [85,86]. Jin reported that NaCl and ABA treatment could promote the occurrence of somatic embryos and increase the expression of stress-related genes in cotton, and the authors speculated that the activation of some stress-response transduction pathways led to the activation of stress-related transcription factors and the expression of downstream stress-related genes in vitro induction [87]. ABA can enhance auxin transport under water stress [88] and regulate proton secretion [89]. The results of our experiment also showed that water treatment alone could induce high gene expression levels. Therefore, water stress may be one of the main factors triggering the induction of cell totipotency. For species in which somatic embryos are not easily induced, we suggest that soaking the explants in acidic water may be beneficial for SE.

Moreover, the auxin gradient and accumulation mediated by auxin transport can regulate proton secretion and H^+^-ATPase activity [90,91,92,93]. Auxin transport enhances the adjustment of proton secretion to aid in the adaptation to water stress. Proton secretion decreased significantly when auxin transport was restrained. Exogenous ABA treatment and water stress promote plasma membrane H^+^-ATPase-mediated proton secretion [89], and the acid-growth hypothesis suggests that the presence of H^+^ in the apoplast is the main reason for cell amplification [64,93,94]. However, after cell wall degradation, protoplasts can survive only in the presence of hormones [95]. Therefore, the intracellular auxin level is a decisive factor in SE. In this experiment, it was determined that the expression levels of *ABCB21* and *PILS7* significantly increased after culture in MS media without the addition of any hormones for 3 days and further increased in the absence of nutrients, especially for *ABCB21* expression. However, these treatments could not induce SE, suggesting that *ABCB21* and *PILS7* may participate in plant water stress adaptation processes by regulating auxin transport and promoting H^+^-ATPase-mediated proton secretion.

**Figure 10:**
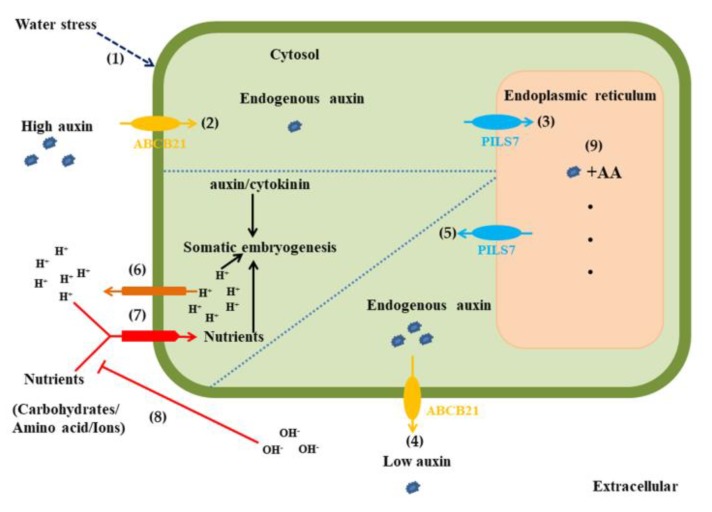
Hypothetical schematic of somatic embryogenesis mediated by auxin transport in *Lilium pumilum*. (1) Water stress activates somatic cells to regain totipotency. (2) ABCB21 transports auxin into the cytosol. (3) PILS7 couples auxin in the endoplasmic reticulum. (4) ABCB21 transports auxin to the extracellular space. (5) PILS7 releases auxin into the cytosol. (6) High H^+^-pumping activity occurs. (7) High H^+^ influx occurs. (8) High pH inhibits nutrient transport. (9) Auxin is coupled with amino acids. Solid arrows indicate transport direction; Dotted arrow indicates exogenous stimulus; T-bars indicates transport suppression.

In summary, *Lilium pumilum* SE may involve two stages (Figure 10). First, water stress activates somatic cells to regain totipotency. Second, totipotent cells develop into somatic embryos at the appropriate ratio of auxin and cytokinin, a suitable pH and adequate nutrition. High exogenous auxin levels and low pH trigger *ABCB21* to transport auxin inward and trigger *PILS7* to couple auxin. Low exogenous auxin and high pH trigger *ABCB21* to transport auxin outward and trigger *PILS7* to release auxin. As a consequence, *ABCB21* and *PILS7* balance the ratio between auxin and cytokinin in cells. Low pH also promotes the entry of nutrients into the cytosol, leading to an increased extracellular pH. Therefore, nutrients inhibit the expression of *ABCB21* and *PILS7*.

## 4. Materials and Methods

### 4.1. Plant Materials and Total RNA Extraction 

**Figure 11:**
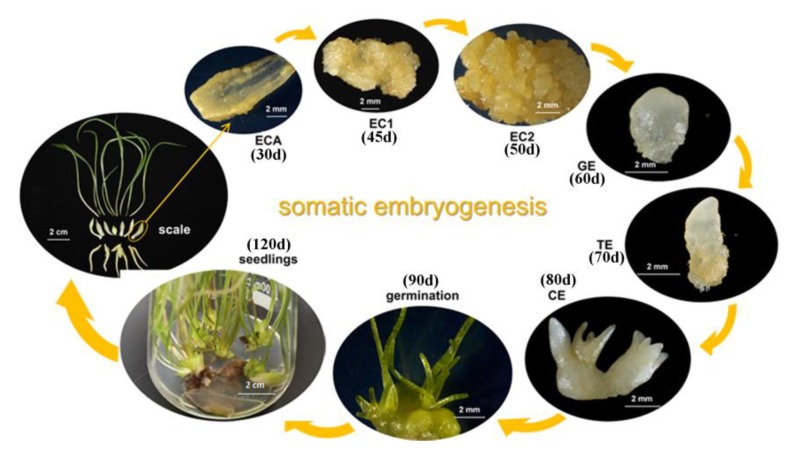
Schematic illustrating the different stages of somatic embryo development in *Lilium pumilum*. ECA, embryonic cells appear; EC1, early proembryo; EC2, late proembryo; GE, globular embryo; TE, torpedo embryo; CE, cotyledon embryo. The time scales of somatic embryogenesis at different stages are shown in the picture. d, days.

The wild *Lilium pumilum* in this study was collected from the southern mountain area of Benxi city, Liaoning province, China and was kept as germplasm resources in our laboratory. The scales of *Lilium pumilum* were used as explants. Scales were cultured in MS media supplemented with 1.0 mg/L PIC for 54 days in darkness. The materials were sampled daily for the first 9 days, sampled every 3 days after 9 days, and sampled every 6 days after 30 days. Roots, stems, leaves and bulbs of germinated plants were sampled. As shown in Figure 11, the sampling process included the following stages: scales; embryonic cells appear (ECA); proembryo (EC1 and EC2); globular embryo (GE); torpedo embryo (TE); cotyledon embryo (CE); germination; and seedings. The samples were frozen in liquid nitrogen and stored at −80 °C for subsequent sequencing and qRT-PCR analysis.

The scales were cultured in different culture media (Table A1). After the scales were cultured in media under different treatment durations, a portion of the scales was then transferred to MS media without hormones for culture for 50 days. There were 30 explants per treatment. Another portion of the scales was frozen in liquid nitrogen and stored at −80 °C for subsequent quantitative analysis. The light culture included 16 h in light and 8 h in darkness each day. The dark culture treatment included darkness throughout the entire experiment.

Total RNA was extracted using the HiPure Plant RNA Mini Kit (Magen), according to the manufacturer’s protocol. The quality and concentration of RNA were detected by using 1.0% agarose gel electrophoresis and an Infinite^®^ 200 Pro (Tecan, Männedorf, Switzerland) microplate reader.

### 4.2. PacBio Library Construction and Sequencing

Total RNA was extracted, and poly-A RNA was enriched. After the poly-A RNA of the different samples was mixed together in equal amounts, cDNA was synthesized by a SMRTbell Template Prep Kit 1.0 (Pacific), after which a SMRTbell library was constructed. The synthesized cDNA was subsequently divided into two libraries, one whose sequences were 1–4 kb and another whose sequences were greater than 4 kb, via agarose gel electrophoresis. The two libraries were separated in accordance with the Pacific Biosciences template preparation and sequencing methods. The two libraries were then sequenced by using a PacBio Sequel sequencer.

### 4.3. Obtaining Full-Length Transcripts and Gene Functional Annotations

SMRT Link software was used to preprocess and filter the raw data to obtain full-length nonchimeric (FLNC) sequences containing primer sequences at both ends and poly-A sequences at the 3’-terminus. The FLNC sequences of the same isoform were clustered and corrected by using iterative clustering and error correction (ICE) within the Cluster module of SMRT Link software to obtain redundant isoform sequences. Non-full-length and chimeric sequences were filtered and removed during FLNC sequences production. The sequences were further corrected and filtered to provide clean isoform sequences. The SGS data (NCBI accession number SRP102354) were obtained from previous research. The polished isoform sequences were further corrected using proovread error correction software. Isoform sequences after error correction were clustered to remove redundancy by cd-hit-est software with the following parameters: -c, 0.99-t, 24-g, 0-al, 0.90-al, 100-as, 0.99-as and 30-m1150. Default values were used for the remaining parameters, and full-length transcription sequences were ultimately obtained. Full-length transcription sequences were compared and annotated with the NR, GO, KOG, KEGG and Swiss-Prot protein sequence databases, and the annotated transcripts were subjected to functional enrichment. The Iso-seq dataset will be uploaded to the NCBI database.

### 4.4. Prediction of the ORFs of Full-Length Transcript Sequences

TransDecoder software release version 2.0.3 (https://github.com/TransDecoder/TransDecoder/wiki) with the default parameters was used to predict the ORFs of the transcripts. To improve the sensitivity of ORF prediction, the predicted protein sequences translated by the ORFs were compared to the information within the Swiss-Prot protein sequence database for homologous protein identification. Protein domains were identified by searching the Pfam database with Hmmscan. The ORFs that were homologous to sequences in known protein libraries or identified as the same protein domain were retained.

### 4.5. Identification and Analysis of Polar Auxin Transport Family Members

Using the coding proteins that predicted the third-generation full-length transcriptome as our data set, we used HMMER3.1 to search the protein sequences containing the ABC domain (PF00005), amino acid transporter (Aa_trans) domain (PF01490) and membrane transport (Mem_trans) domain (PF03547). PfamScan (*e*-value < 0.001) (https://www.ebi.ac.uk/Tools/pfa/pfamscan/) was used to analyze the predicted protein domains, and ExPASY (https://web.expasy.org/protparam/) was used to analyze the pI and MW of the proteins. The sequence information of the ABC transporter superfamily members, auxin efflux carrier family members and amino acid/polyamine transporter 2 family members of *Arabidopsis thaliana* were downloaded from the UniProtKB/Swiss-Prot database. The members of the three families in *Lilium pumilum* and *Arabidopsis thaliana* were compared by using MAFFT [96]. FastTree [97] was used to construct an evolutionary tree. iTOL release version 5.1.1 (https://itol.embl.de/itol.cgi) was used to draw the evolutionary tree. MEME release version 5.1.0 (http://meme-suite.org/tools/meme) was used to analyze the conserved domains of the family members.

### 4.6. Cloning and Expression Pattern Analysis of Genes Involved in Polar Auxin Transport

To verify the accuracy of the full-length transcriptome data and screen the key genes involved in SE, the genes identified to be involved in polar auxin transport were cloned, and their expression patterns were analyzed. First, specific primers for cloning and qRT-PCR were designed. One microgram of total RNA from each sample was reverse transcribed into cDNA by using Moloney Murine Leukemia Virus (M-MLV) reverse transcriptase (Promega, Madison, USA) in a 20 µL system. PrimeSTAR MAX DNA Polymerase (Takara, Dalian, China) was used for gene cloning. qRT-PCR was conducted via SYBR Green Master Mix on a QuantStudio 3 system (Life Technologies, San Francisco, CA, USA). The total RNA (1.6 µg) was added to a 50 µL system, and RNA samples from different SE stages were reverse transcribed via PrimeScript™ RT Master Mix (Takara, Dalian, China). The reaction procedure was as follows: 37 °C for 30 min followed by 85 °C for 5 sec Then, TB Green^®^ Fast qPCR Mix (Takara, Dalian, China) was used for quantitative analysis (20 µL). *FP* was used as the internal reference gene [98], and the relative expression level of the genes was calculated according to the 2^−^^△△Ct^ method, with three biological replicates and three technical replicates. The expression variation was calculated by the mean expression value of the gene in a given treatment divided by the mean expression value of the gene in the control treatment. Genes whose expression variation was greater than two-fold greater than that in the control treatment were considered significantly upregulated, those whose expression variation was less than 0.5-fold were considered significantly downregulated, and those whose expression variation was greater than 0.5-fold but less than two-fold greater were considered not significantly different.

## Figures and Tables

**Table 1:** The effects of picloram (PIC) and light on somatic embryo induction (50 days).

Treatment	PIC Treatment Duration(days)	Culture Duration in MS(days)	Inductivity of Callus (No.)	Inductivity of Buds (No.)	No. of Roots
Light culture	3	47	80.0% (24)	50.0% (47)	18
	6	44	90.0% (27)	50.0% (41)	25
	9	41	96.7% (29)	36.7% (39)	18
	12	38	100% (30)	30.0% (29)	20
	15	35	100% (30)	26.7% (20)	5
	18	32	100% (30)	26.7% (20)	19
	21	29	100% (30)	20.0% (15)	15
	50	0	100% (30)	13.3% (4)	0
Darkness culture	3	47	33.3% (10)	10.0% (8)	12
	6	44	43.3% (13)	10.0% (4)	2
	9	41	66.7% (20)	10.0% (10)	5
	12	38	80.0% (24)	16.7% (11)	15
	15	35	96.7% (29)	13.3% (6)	18
	18	32	100% (30)	10.0% (9)	23
	21	29	100% (30)	3.3% (1)	26
	50	0	100% (30)	0	0

**Table 2:** The results of callus induction in different media.

Treatment	M	P1	P2	P3	P4	P5	P6	pH1	pH2	pH3	pH4	pH5	A1	A2	A3	A4
No. of callus	2	15	18	15	9	7	3	17	18	2	0	0	2	15	12	12
Inductivity/%	11.1	83.3	100	83.3	50.0	38.9	16.7	94.4	100	11.1	0	0	11.1	83.3	66.7	66.7
No. of buds	3	28	16	7	0	0	0	0	0	0	3	0	0	0	0	0

Note: M, MS media; P1–P6, MS media supplemented with PIC at 0.5, 1.0, 2.0, 3.0, 4.0 and 5.0 mg/L, respectively; pH1–pH5, MS media supplemented with PIC at 1.0 mg/L with the pH adjusted to 4.8, 5.8, 6.8, 7.8 and 9.8, respectively; A1, MS media without macroelements but supplemented with PIC at 1.0 mg/L; A2, MS media without microelements but supplemented with PIC at 1.0 mg/L; A3, MS media without iron salts but supplemented with PIC at 1.0 mg/L; A4, MS media without organic substances but supplemented with PIC at 1.0 mg/L; all the media were adjusted to pH = 5.8 with 1 M NaOH except pH1 and pH3–pH5.

## Supplementary Materials

Supplementary materials can be found at https://www.mdpi.com/1422-0067/21/2/453/s1.

## Abbreviations

MS (Culture medium)	Murashige-Skoog
PIC (IAA analogue)	Picloram (Sigma-Aldrich, USA)
6-BA (Cytokinin)	6-Benzylaminopurine (Tiangen, Beijing, China)
TIBA (Auxin transport inhibitors)	2,3,5-triiodobenzoic acid (Sigma-Aldrich, USA)

## Appendix A

**Table A1:** The media component for different treatment.

Treatment	PIC (mg/L)	6-BA (mg/L)	TIBA (mg/L)	MS	pH	Duration (days)	Light/Dark
D1-D7	1.0			√	5.8	3, 6, 9, 12, 15, 18, 21	Dark
L1-L7	1.0			√	5.8	3, 6, 9, 12, 15, 18, 21	Light
M				√	5.8	3, 50	Light
P	1.0			√	5.8	3, 50	Light
B		1.5		√	5.8	3, 50	Light
P+T	1.0		1.0	√	5.8	3, 50	Light
P1	0.5			√	5.8	3, 50	Light
P2	1.0			√	5.8	3, 50	Light
P3	2.0			√	5.8	3, 50	Light
P4	3.0			√	5.8	3, 50	Light
P5	4.0			√	5.8	3, 50	Light
P6	5.0			√	5.8	3, 50	Light
A1	1.0			no macro	5.8	3, 50	Light
A2	1.0			no micro	5.8	3, 50	Light
A3	1.0			no iron salts	5.8	3, 50	Light
A4	1.0			no organic	5.8	3, 50	Light
pH1–pH5	1.0			√	4.8/5.8/6.8/7.8/9.8	3, 50	Light
