# Supplementary material for: Full-Length Transcriptome Analysis of the ABCB, PIN/PIN-LIKES, and AUX/LAX Families Involved in Somatic Embryogenesis of Lilium pumilum DC. Fisch
Source: Int J Mol Sci. 2020 Jan 10;21(2):453. doi: 10.3390/ijms21020453 (PMC7014436; doi:10.3390/ijms21020453)
Supplement: Supplementary file 1 [file ijms-21-00453-s001.pdf]

Supplementary Files

Full-length Transcriptome Analysis of the *ABCB*, *PIN*/*PIN-LIKEs*, and *AUX*/*LAX* Families Involved in Somatic Embryogenesis of *Lilium pumilum* DC. Fisch.

Shengli Song [1], Zhiping Wang [1], Yamin Ren [1], Hongmei Sun [1,2]*

[1] Key Laboratory of Protected Horticulture of Education Ministry and Liaoning Province, College of Horticulture, Shenyang Agricultural University, Shenyang 110866, China; ssl_syau@163.com (S.S.); wangzp6119@163.com (Z.W.); RYM1258252544@163.com (Y.R.)

[2] National and Local Joint Engineering Research Center of Northern Horticultural Facilities Design and Application Technology, Horticulture Department, Shenyang Agricultural University, Shenyang 110866, China

* Correspondence: hmbh@sina.com; sunhm@syau.edu.cn; Tel.: +8624-88487143

Table S1: The Comparison of Transcript Length and Number

Sample	Total	Total	Average	Maxnum	Minnum	Median	N50
Iso-seq	241,308,336	119,649	2,017	13,678	123	1,376	3,406
RNA-seq	93,826,445	123,284	761	12,365	205	495	1,149

Table S2: The ABC and PIN/PILS and AUX/LAX Family in *Lilium pumilum*

Group	Transcript ID	Names	Topology	Len(aa)	MW(Kd)	pI
Subfamily A	i5_LQ_sampleda152d|c726/f1p0/5987	LpABCA1	$(T_2MD_3\text{-NBD})_2$	1913	213.75	7.88
	i4_HQ_sampleda152d|c7936/f2p3/4056	LpABCA2	T_2MD_3-NBD	617	68.25	7.09
	i3_LQ_sampleda152d|c15709/f1p0/3122	LpABCA8	T_2MD_3-NBD	609	68.33	7.61
	i0_LQ_sampleda152d|c273499/f1p0/925	LpABCA12	NBD-DUF4162	253	28.16	8.33
Subfamily B	i4_HQ_sampleda152d|c1964/f3p0/4463	LpABCB1	$TMD\text{-}NBD_2$-TMD-NBD	1418	154.36	8.41
	i4_HQ_sampleda152d|c31031/f11p2/4008	LpABCB2	$(TMD\text{-}NBD)_2$	1269	139.37	8.74
	i3_LQ_sampleda152d|c207431/f1p0/3059	LpABCB4	TMD-NBD	670	72.68	6.02
	i4_LQ_sampleda152d|c10135/f1p2/4672	LpABCB6	$(TMD\text{-}NBD)_2$	1401	155.22	6.31
	i3_HQ_sampleda152d|c19025/f3p0/3634	LpABCB8	TMD-NBD-TMD	913	100.93	8.42
	i1_LQ_sampleda152d|c210904/f1p0/1944	LpABCB9	NBD	324	35.04	5.58
	i1_LQ_sampleda152d|c270294/f1p22/1888	LpABCB10	TMD-NBD	589	64.96	9.12
	i3_LQ_sampleda152d|c99644/f1p0/3482	LpABCB13	$(TMD\text{-}NBD)_2$	1018	111.27	8.83
	i3_HQ_sampleda152d|c6437/f2p0/3697	LpABCB14	TMD-NBD	605	67.08	8.87
	i7_LQ_sampleda152d|c92/f1p0/7819	LpABCB19	$(TMD\text{-}NBD)_2$	1260	137.77	8.76
	i4_HQ_sampleda152d|c2655/f2p6/4384	LpABCB21	$(TMD\text{-}NBD)_2$	1300	140.89	8.48
	i2_LQ_sampleda152d|c4516/f1p0/2627	LpABCB25	TMD-NBD	752	82.85	9.18
	i1_LQ_sampleda152d|c221975/f1p0/1920	LpABCB26	TMD-NBD	567	63.41	6.69
	i1_HQ_sampleda152d|c23704/f2p0/1972	LpABCB28	TMD-NBD	609	65.95	8.55

	i4_LQ_sampleda152d|c24216/f1p2/4055	LpMDR	(TMD-NBD)$_2$	1234	133.88	8.73
Subfamily C	i1_HQ_sampleda152d|c14282/f4p41/1344	LpABCC1	NBD	358	40.43	5.42
	i5_HQ_sampleda152d|c2892/f77p0/5268	LpABCC2	(TMD-NBD)$_2$	1627	183.66	7.05
	i4_LQ_sampleda152d|c3534/f1p0/4925	LpABCC3	(TMD-NBD)$_2$	1466	163.16	6.34
	i4_LQ_sampleda152d|c34384/f1p0/4175	LpABCC4	(TMD-NBD)$_2$	1294	144.24	5.82
	i4_HQ_sampleda152d|c10798/f3p0/4928	LpABCC5	(TMD-NBD)$_2$	1521	169.22	8.59
	i4_HQ_sampleda152d|c31276/f48p8/4943	LpABCC9	(TMD-NBD)$_2$	1524	170.51	8.61
	i4_HQ_sampleda152d|c9995/f3p0/4632	LpABCC10	(TMD-NBD)$_2$	1456	162.14	8.19
	i3_LQ_sampleda152d|c75804/f1p0/3713	LpABCC13	NBD	266	29.35	5.37
	i4_HQ_sampleda152d|c31509/f20p0/4862	LpABCC14	(TMD-NBD)$_2$	1481	164.86	6.78
	i5_LQ_sampleda152d|c2349/f1p4/5173	LpMRP4	TMD-NBD	714	79.36	8.93
Subfamily D	i4_LQ_sampleda152d|c8298/f1p13/4870	LpABCD1	(TMD$_2$-NBD)$_2$	1339	150.23	9.26
	i2_HQ_sampleda152d|c1770/f2p0/2587	LpABCD2	TMD$_2$-NBD	724	80.89	6.04
Subfamily E	i2_LQ_sampleda152d|c2634/f1p0/2604	LpABCE2	RLI-Fer4-NBD-NBD	605	68.29	7.48
Subfamily F	i2_LQ_sampleda152d|c11548/f1p10/2009	LpABCF1	NBD-NBDx-NBD	593	66.60	6.34
	i3_LQ_sampleda152d|c121904/f1p0/3214	LpABCF3	NBD-NBDx-NBD	717	79.80	5.74
	i2_HQ_sampleda152d|c1673/f2p1/2379	LpABCF4	NBD-NBD	738	81.33	5.90
	i2_HQ_sampleda152d|c321/f7p0/2309	LpABCF5	NBD-NBDx-NBD	700	78.57	8.42
Subfamily G	i2_HQ_sampleda152d|c1818/f2p0/2594	LpABCG1	NBD-T$_2$MD	757	83.20	9.30
	i4_HQ_sampleda152d|c30056/f5p1/4744	LpABCG2	NBDn-NBD-T$_2$MD-Pa-NBD-T$_2$MD	1475	167.46	8.62
	i4_HQ_sampleda152d|c16104/f4p0/4760	LpABCG3	NBDn-NBD-T$_2$MD-Pa-NBD-T$_2$MD	1450	164.26	8.23
	i2_HQ_sampleda152d|c427/f4p1/2209	LpABCG7	NBD-T$_2$MD	667	73.43	9.28
	i2_LQ_sampleda152d|c8246/f1p3/2288	LpABCG11	NBD-T$_2$MD	723	80.37	8.86
	i2_LQ_sampleda152d|c6925/f1p0/2206	LpABCG20	NBD-T$_2$MD	732	80.05	9.24
	i2_LQ_sampleda152d|c13526/f1p0/2686	LpABCG22	NBD-T$_2$MD	743	82.28	9.22
	i3_LQ_sampleda152d|c136968/f1p0/3705	LpABCG28	NBD	1098	120.87	9.18
	i4_HQ_sampleda152d|c31081/f11p0/4568	LpABCG31	NBDn-NBD-T$_2$MD-Pa-NBD-T$_2$MD	1418	160.98	8.29
	i3_LQ_sampleda152d|c85679/f1p0/3243	LpABCG34	T$_2$MD-Pa-NBD	610	68.36	7.64
	i4_LQ_sampleda152d|c10792/f1p0/4478	LpABCG35	NBDn-NBD-T$_2$MD-Pa-NBD-T$_2$MD	1427	161.19	8.17
	i4_LQ_sampleda152d|c7886/f1p4/4657	LpABCG36	NBDn-NBD-T$_2$MD-Pa-NBD-T$_2$MD	1449	164.09	8.54
	i4_LQ_sampleda152d|c4008/f1p0/4472	LpABCG37	NBDn-NBD-T$_2$MD-Pa-NBD	1009	112.94	8.63
	i4_HQ_sampleda152d|c31176/f25p2/4629	LpABCG39	NBDn-NBD-T$_2$MD-Pa-NBD-T$_2$MD	1479	166.35	8.74
	i4_LQ_sampleda152d|c10977/f1p0/4572	LpABCG42	NBDn-NBD-T$_2$MD-Pa-NBD-T$_2$MD	1518	172.30	8.68
	i4_HQ_sampleda152d|c3065/f2p0/4249	LpABCG44	T$_2$MD-Pa-NBD	756	85.36	8.96
	i4_LQ_sampleda152d|c21410/f1p7/4437	LpABCG48	NBD-T$_2$MD-Pa-NBD	1027	115.00	8.01
	i4_LQ_sampleda152d|c13344/f1p0/4565	LpABCG51	T$_2$MD-Pa-NBD-T$_2$MD	1142	128.59	8.75
	i3_LQ_sampleda152d|c78733/f1p0/3632	LpABCG53	T$_2$MD-Pa-NBD-T$_2$MD	1082	123.03	8.78
	i4_HQ_sampleda152d|c3015/f3p0/4645	LpTUR2	NBDn-NBD-T$_2$MD	769	86.67	8.58
Subfamily I	i1_LQ_sampleda152d|c123242/f1p0/1079	LpABCI1	NBD	253	28.74	9.99
	i1_LQ_sampleda152d|c120814/f1p2/1459	LpABCI6	NBD	325	35.05	6.98
	i1_LQ_sampleda152d|c358086/f1p2/1199	LpABCI11	NBD	270	29.82	9.21

	i1_HQ_sampleda152d|c281486/f3p0/1073	LpABCI19	NBD	295	32.85	6.34
	i1_HQ_sampleda152d|c394849/f10p1/1197	LpABCI20	NBD	353	38.77	6.97
	i1_LQ_sampleda152d|c232559/f2p0/1017	LpSTAR1	NBD	289	31.20	8.29
PIN1	i2_HQ_sampleda152d|c375/f6p0/2267	LpPIN1A	Mem_trans	619	66.84	9.06
	i1_HQ_sampleda152d|c15063/f5p2/1033	LpPIN1C	Mem_trans	287	30.39	9.24
	i1_LQ_sampleda152d|c264634/f1p1/1048	LpPIN1D	Mem_trans	258	27.26	9.30
PIN2	i1_LQ_sampleda152d|c14246/f1p1/1188	LpPIN2	Mem_trans	306	33.58	9.76
PIN3	i1_HQ_sampleda152d|c4662/f2p1/1443	LpPIN3	Mem_trans	377	39.93	6.93
	i2_LQ_sampleda152d|c4634/f1p0/2187	LpPIN3A	Mem_trans	651	69.88	7.21
PIN5	i1_LQ_sampleda152d|c166345/f1p0/1301	LpPIN5	Mem_trans	221	24.23	9.12
PIN-LIKES	i1_HQ_sampleda152d|c328920/f19p3/1514	LpPILS2	Mem_trans	458	50.35	8.37
	i1_LQ_sampleda152d|c9088/f2p1/1306	LpPILS5	Mem_trans	335	36.24	7.22
	i1_LQ_sampleda152d|c218219/f1p0/1715	LpPILS6	Mem_trans	467	50.66	9.05
	i1_HQ_sampleda152d|c393793/f3p3/1723	LpPILS7	Mem_trans	422	45.86	6.13
Auxin transporter	i1_LQ_sampleda152d|c66045/f1p2/1025	LpAUX1	Aa_trans	240	27.13	9.54
	i2_LQ_sampleda152d|c9931/f1p0/2085	LpLAX2	Aa_trans	495	55.22	8.43
	i1_LQ_sampleda152d|c29598/f1p0/1660	LpLAX3	Aa_trans	473	53.29	9.22
Amino acid permease	i1_HQ_sampleda152d|c3837/f2p0/1243	LpAAP1	Aa_trans	350	38.85	8.49
	i1_LQ_sampleda152d|c46289/f1p1/1727	LpAAP2	Aa_trans	517	56.77	8.50
	i1_HQ_sampleda152d|c202328/f2p3/1828	LpAAP3	Aa_trans	490	53.55	8.83
	i1_HQ_sampleda152d|c41926/f4p0/1697	LpAAP6	Aa_trans	485	53.66	8.05
	i1_HQ_sampleda152d|c69950/f2p2/1617	LpAAP7	Aa_trans	457	50.70	8.30
Amino acid transporter	i1_LQ_sampleda152d|c26748/f1p2/1774	LpAvt1A	Aa_trans	530	57.67	5.61
	i0_LQ_sampleda152d|c12760/f1p0/1026	LpAvt1C	Aa_trans	274	29.19	8.63
	i1_LQ_sampleda152d|c213746/f1p1/1491	LpAvt3	Aa_trans	408	43.77	6.49
	i0_LQ_sampleda152d|c75545/f1p0/721	LpAvt3B	Aa_trans	222	24.20	4.88
	i1_HQ_sampleda152d|c19391/f6p2/1556	LpAVT6A	Aa_trans	467	50.19	7.78
	i1_LQ_sampleda152d|c270480/f1p0/1669	LpAvt6B	Aa_trans	212	22.66	6.82
	i1_HQ_sampleda152d|c12767/f2p0/1591	LpAVT6C	Aa_trans	436	46.59	6.77
	i2_LQ_sampleda152d|c4112/f1p1/2133	LpAvt6E	Aa_trans	465	49.65	6.60
	i1_LQ_sampleda152d|c9961/f1p0/1600	LpANT1	Aa_trans	438	46.64	6.59
GABA transporter	i1_HQ_sampleda152d|c9264/f2p0/1527	LpGAT1	Aa_trans	496	53.82	8.56
	i3_LQ_sampleda152d|c23982/f1p0/3566	LpGAT2	Aa_trans	302	33.26	7.83
Proline transporter	i1_HQ_sampleda152d|c394756/f2p0/1540	LpPROT1	Aa_trans	459	51.75	9.49
	i1_HQ_sampleda152d|c10044/f2p0/1583	LpPROT2	Aa_trans	459	49.87	8.79
Lysine histidine transporter	i1_LQ_sampleda152d|c82284/f1p0/1846	LpLHT1	Aa_trans	490	54.53	9.07
	i1_HQ_sampleda152d|c9176/f2p1/1739	LpAATL1	Aa_trans	522	56.97	9.59

Note: NBD=ABC_tran; NBDn=ABC_trans_N; NBDx=ABC_tran_Xtn; TMD=ABC_membrane; Len=Length; MW=Molecular weight; pI=Isoelectric point

Table S3: The Primers for Genes Cloning

Names	Primers	Sequences	Product length (bp)
B1	ABCB1-F	CTGTGTGCATTGAGTTTGCG	4222
	ABCB1-R	ACCAGCCTTATGTAAATCTCTCTT	
B2	ABCB2-F	CAACGCAGAGTACATGGGGGT	3850
	ABCB2-R	CACGAAAGTCGCCACTGTGC	
B6	ABCB6-F	GACCAGCGAAGAAAAGACGG	4459
	ABCB6-R	ACCATCAACCAACCTGTGAGTATC	
B19	ABCB19-F	CGGCTCGACTGGCTTCTCAT	3768
	ABCB19-R	CCCATCCCTCGAACATATCTACAT	
B21	ABCB21-F	GCCGTAATGCTTGCCCTCTAAAT	4084
	ABCB21-R	TGAAGAGGCTTTTGAGTGAAGTGCT	
B15	ABCB25-F	GCCCCACGAGGGTTTCCAAT	2411
	ABCB25-R	CGCCTACTGGCTTCCATCTCAAC	
MDR	MDR-F	CCACAACCCCTGTAACAA	3806
	MDR-R	CTTCATTCTCTGATACCACTCCAT	
P1	PIN1A-F	CCCCCATCCGCAAATCAATAC	2092
	PIN1A-R	CAATCAAGTCCGTATCATACCCAAAC	
P3	PIN3A-F	GCAAACTGTAATCTCCCCTCAG	2112
	PIN3A-R	GGCTAACTACTGATTTCCGAGG	
S2	PILS2-F	ACAGAGGGAGGGAGAGAACG	1470
	PILS2-R	CTAACAAGAAACACCAAAGTCACAC	
S6	PILS6-F	GAGGTGTTTGTTCGCTGTTGTC	1362
	PILS6-R	ACCATCTCTCCACGCCTTGT	
S7	PILS7-F	GAAAAACCTCTGCTTGGTGTTATTG	1462
	PILS7-R	AAATCATCTTCCATTCCCTCCCT	
L2	LAX2-F	CTCCAACTCTCTCTCACCCAACC	1822
	LAX2-R	CAGCACAGACCTATCCAAACCTAAC	
L3	LAX3-F	TAGTTTACGCAGAGATGGCATCCG	1429
	LAX3-R	ACTCTAACCCAGTCCTAAACCTTGCG	

Table S4: The Primers for Real Time PCR

Primers	Sequences	Product length	TM
ABCB1-QF	GCGGGTGTTGATAGACGGCA	131	60
ABCB1-QR	CCCTCCCGTAAGCAATGTTGTC		
ABCB2-QF	CAGTTCAGTTACCCCTCACGC	107	56
ABCB2-QR	TTCCCAGACCCACTTCCAC		
ABCB6-QF	GCCGCCCACCGAATAAGTT	158	62
ABCB6-QR	GCCGAAAATAGCAGCACCAATAC		
ABCB19-QF	GCTGGGGAGGGAGTGAGTAAC	149	60
ABCB19-QR	TGCGAGAGCCCGTAGAGAAG		
ABCB21-QF	CCGTAGGATTGGGTCTTGGT	108	60
ABCB21-QR	TGGCACCAGTGTATCCTTTATCTA		
ABCB25-QF	CGCTTAGAGCCATCCGCACT	143	56
ABCB25-QR	CGAAAGAATGAAGTTTATTGCCCG		
MDR-QF	TACTAAGAGGGTGCGAGAGCG	184	62
MDR-QR	GATGACGACGGCAGAGATGG		
PIN1A-QF	TCAAGTGGTGGCACATCTTCTCT	126	58
PIN1A-QR	GGAGGTTCATCGTGTAGGGGT		
PIN3A QF	CTCGCCACGCTTCCTAACAC	105	58
PIN3A QR	GCACTGGAGAACAACGACCTG		
PILS2-QF	CTTCATCGCCAGAATCTTTGC	150	58
PILS2-QR	CTGATTCGTCGGACAACTTTAGG		
PILS6-QF	TCGTGATTGGTGTTACTCCATTTC	136	60
PILS6-QR	AGATTACCACCCAAAGCGAGC		
PILS7-QF	TCAACAGATGAAAAGACGATACCG	155	60
PILS7-QR	GCGCCAACAATTAAGCCG		
LAX2-QF	AAGAAGATGAACCCTCGGATG	149	58
LAX2-QR	CGAGTATGGCAGCGTCAGTAG		
LAX3-QF	CGTGTGGGAGAAAGCAATCG	113	58
LAX3-QR	AAGGGGAAGATGATGGCGAG		
